# Baubles, Bangles, and Biotypes: A Critical Review of the use and Abuse of the Biotype Concept

**DOI:** 10.1673/031.010.14136

**Published:** 2010-10-11

**Authors:** D. A. Downie

**Affiliations:** Department of Zoology and Entomology, Rhodes University, Grahamstown, 6140 South Africa

**Keywords:** biological races, genetic variation, host plant resistance, host races, phenotypic variation, population differentiation, strains

## Abstract

Pest species of insects are notoriously prone to escape the weapons deployed in management efforts against them. This is particularly true in herbivorous insects. When a previously successful tactic fails the insect population has apparently adapted to it and is often considered to be a new or distinct entity, and given the non-formal category ‘biotype’. The entities falling under the umbrella term ‘biotype’ are not consistent either within or between biotypes, and their underlying genetic composition and origins, while generally unknown, are likely heterogeneous within and variable between biotypes. In some cases race or species may be more appropriate referents. Some examples of applications of the concept in the context of host plant resistance are discussed. It is argued here that the term ‘biotype’ and its applications are overly simplistic, confused, have not proved useful in current pest management, and lack predictive power for future management.

## Introduction

**bauble** (bô'bel) *n.* 1. A small, showy ornament of little value, a trinket. 2. *Archaic.* A baton carried by a court jester as a mock scepter of his office. (The American Heritage Dictionary)

**bangle** [băng'gel] An ornament that hangs from a bracelet or necklace. (The American Heritage Dictionary)

**biotype** [bi'ō-tīp] a taxonomic concept mostly used by non-taxonomists [Eastop 1973]

Phenotypic variation is ubiquitous in natural populations and its description is a fundamental occupation of ecology and evolutionary biology. Understanding the nature of phenotypic variation requires a description of the genetic and environmental variation that causes it, which requires consideration of the hierarchic structure of alleles within loci, genes within individuals, individuals within populations, and populations within species. Until recently, systematics treated only that part of the hierarchy above the species level. Variation among individuals within populations has always been the focus of population genetics. Increasingly, differentiation among populations at the intraspecific level has been seen as an important topic for research in understanding biodiversity, speciation, and adaptive change (Avise et al. 1987; Avise 2000). Entomologists working on pest species that vary in their responses to the methods used to control them often discuss their results in terms of population level differences. Many of these researchers have seen fit to name or classify the variants they find under the pseudo-taxonomic category ‘biotype’, generally naming these ‘taxa’ with letters or numbers. It has not been at all clear, however, if the variants so named represent genetically based phenotypic variation due to allelic, genotypic, population, or species level differences. Since allelic and genotypic variations are transient properties of the individuals in populations, and species level differences are the domain of systematics, it is only population level differences where intraspecific categories such as biotype can have any meaning. Because of the potential for learning more about the transitional steps toward speciation, it is important to distinguish population differentiation from other forms of intraspecific variation.

Though controversial, the practice of describing and naming biotypes continues. The biotype concept and its precursors have been reviewed a number of times (Thorpe 1930; Smith 1941; Eastop 1973; Claridge and den Hollander 1983; Diehl and Bush 1984; Saxena and Barrion 1987), but its continued use and the entry of a new generation of entomologists into the scientific community warrants a new critical review. A major thesis of this review will be that the biotype concept has led to simplistic, and as a consequence, misleading perceptions of the variation found within and among pest populations. Its use has also impeded progress in understanding the true nature and consequences of that variation and in formulating the appropriate responses to it. The problem is homologous to the ‘species problem’. As in that conundrum, entomologists would like to be confident that the entities they name or refer to have a real existence. If they do not, there is a risk of severely misinterpreting nature. Species may or may not be discrete entities — as an intraspecific category, biotypes are certainly *not* discrete entities. For transitional phases in the process of divergence and differentiation, the members of which have distinct traits or distributions of character states, it may be both practically useful and scientifically meritorious to categorize and assign at least informal names or coding. Do named biotypes represent such entities? If so, is not race a far less ambiguous term to apply? If not, what is being named or referred to?

After an exposition of the problem that the use of the biotype concept poses, its use will be placed in perspective by briefly reviewing its history, and an attempt will be made to clarify the use of some associated terms and concepts. Then some simple aspects of population genetics theory that relate to phenotypic variation will be outlined. Finally some notable examples of application of the concept will be briefly reviewed. The discussion will focus on herbivorous insects and host plant resistance (HPR), but the conclusions may be generalized to any attempts to name, categorize, or classify organisms based on a single ecological or physiological criterion, such as resource use (i.e. weed susceptibility/resistance to biocontrol agents, insecticide resistance, or virus transmission ability). It is noted that the term has been applied more broadly by many entomologists, the best example of which would be in the whitefly, *Bemisia tabaci.*

### The nature of the problem

To the dismay of entomologists and plant breeders, insects seem more often than not to be able to overcome the armaments thrown at them in the form of resistant host plants. When this occurs, with distressing regularity it is declared that a ‘new type’ of insect has arisen, or *developed.* However, it is seldom (or never) the case that the original population of insects was fully characterized from either an ecological or genetic standpoint, so it is never known exactly what, if anything, is ‘new’ about the insects damaging resistant plants. A crop cultivar that was not damaged previously is now damaged and the phenomenon (the ‘new type’ of insect) is deemed worthy of being named, or at least referred to as a new entity, a ‘biotype’. In this case the act of classification is based on a partially measured single, but compound trait (insect context-dependent performance, or its consequence, plant damage) at a single point in time, and the concordances of the classification with the genealogical relationships of included individuals and underlying component traits among included individuals are not known and are seldom considered.

Grouping objects into sets can be performed on the basis of any shared characteristic of the objects that suits one's purposes, however, when the objects are biological entities any grouping that uses superficial or artificial characters will lead to a misclassification and a resultant failure in predictive power. There is, in fact, a well-established criterion in biology for determining ‘true’ relationship, namely common descent, and it is the only valid basis for naming groups of organisms. This is the domain of phylogenetic systematics, but the importance of grouping organisms that are truly related (monophyletic) is important at the intraspecific level as well if the goals are to deploy and predict responses to management tactics. In the context of insect performance on any given host plant, the future performance on new host plants, or on the same hosts at a different time or place may differ when a given phenotype is produced by different genotypes. A similar statement could be made regarding any other biological parameter. Genotype here is meant to imply the genetic constitution at either a single locus, multiple loci, or in the genetic background. There is growing recognition that reaction norms may vary among genotypes within a population as well as among them (Schlichting and Pigliucci 1998). The reaction norm is not fully characterized by measuring it in only two or a few environments (host plants in this discussion). Consequently, unless the genetic constitution is the same among the individuals being categorized a new environment may well produce a different set of phenotypes.

Survival and reproduction on a plant is determined by multiple factors that are extrinsic as well as intrinsic to the interaction. The compound trait of fitness can be decomposed into behavioral, physiological, and morphological traits; and fitness consequences can arise from changes in any of these components. Each may be subject to inter-individual variation in exactly the same manner as the more conventional morphological traits such as tibia length, number of abdominal bristles, interocular distance, and hair color or taste preferences in humans. The same phenotype may arise from adaptation to physiological factors or from behavioral cues, for example. Most often, when testing herbivorous insects on resistant or susceptible host plants, entomologists measure performance in terms of survivorship, developmental time, fecundity, and often an index of plant damage. That multiple routes may be taken to reach a given level of performance (or damage) is acknowledged, but the implications of this for how the differences or similarities among tested insects should be interpreted are not. The functional and operational utility of categorizing the insects showing a given phenotype is thought to be self-evident. Given the mandate to devise or inform strategies for deploying resistant host plants, and that the result of herbivore survival and reproduction is crop damage, this simplification of a complex process may be understandable, if not very productive in the long term (or even the short term). At issue is whether the perception and subsequent grouping of potentially unrelated genotypes as a unitary entity, a biotype, or the splitting of an otherwise homogenous group has any predictive power under conditions other than the one that was originally used to define the grouping. As such, it is an issue central to scientific induction and inference.

### History and definitions of the term biotype

#### Meaning what we say, saying what we mean

Walsh (1864) was an early proponent of the idea that host associated phenotypic variation in herbivorous insects could lead to population structuring and divergence, and have potential consequences for the taxonomy of such insects. Early reviews of host associated variation in performance of insects on crop plants (Thorpe 1930; Smith 1940) were essentially discussing many cases that can now be understood to fall, more or less, under the concept of a biotype. Though Dobzhansky in his seminal work, *Genetics and the Origins of Species* (1937), had used the term biotype in reference to asexual organisms that “clustered around some of the adaptive peaks in the field of gene combinations”, it was apparently not until Printz (1937) and Painter (1941) that the term biotype, without definition, was applied to situations where insects differed in their responses to crop plants deployed for their resistance to insect feeding. A thorough discussion of biotypes was given in Painter's excellent book (1951) in which he freely interchanges ‘biotype’ with ‘biological strains’ and ‘biological races’. Since then, a number of definitions, both formally and informally, have been proposed. Some of these follow:

*“…populations that are able to reproduce and survive on …cultivars developed for resistance to this insect, or are able to resist …insecticides”* (Nielson et al. 1970, p. 1822)

*“…the terms “race” and “biotype” are interchangeable and are defined as one or more Hessian flies that have specific phenotypes with respect to their ability or inability to survive on and stunt wheats having specific genes for resistance.* (Sosa and Gallun 1973, p. 1065)

*“A biotype… is an individual or a population whose phenotype is determined by the interaction between…plants having different genes for resistance and the larvae 's ability or inability to survive on and stunt the…plant.”* (Gallun 1978, p. 481)

*“…an individual or population that is distinguished from the rest of its species by criteria other than morphology, for example, a difference in parasite ability.”* (Gallun and Khush 1980, p.67)

*“…pest populations which differ in their ability to infect rice varieties with specific major genes for resistance.”* (Saxena and Barrion 1983, pp. 54)

*“Biotypes are most commonly entomophagous or phytophagous parasites or parasitoids distinguished by survival and development on a particular host or by host preference for feeding, oviposition, or both. …diverse biological differences have been used to designate populations as biotypes in* *the literature.”* (Diehl and Bush 1984, pp. 471–472)

*“Broadly considered, the term biotype is an intraspecific category referring to insect populations of similar genetic composition for a biological attribute. The biotype populations may be partially and temporarily sympatric, allopatric or parapatric with other compatible populations, but differ in one or more biological attributes. ”* (Saxena and Barrion 1987, p. 454)

*“Strain designates a population arising from a single collection or clonal individual; biotype is a category designating shared phenotypic traits; host race is a biotype that is better adapted to a specific host than are other biotypes. ”* (Granett et al. 2001, p. 400)

*“ ‘Biotype’ is a taxonomic concept mostly used by non-taxonomists and has been defined as consisting of all individuals of equal genotype.” op. cit.*

*“Biotype' is a taxonomic concept mostly used by non-taxonomists and has been defined as consisting of all individuals of equal genotype. Biotypes are recognized by a biological function rather than by morphological characters. In practice a biotype contains those individuals performing whatever biological feat interests the observer and thus may contain one or more races or strains.”* (Eastop 1973, p. 40)

All these definitions agree that categorization is based on differential performance on different hosts or in some other biological attribute, but there is internal inconsistency within and across definitions as to what level of the hierarchy is actually being considered. Diehl and Bush do not specify any particular level of inclusiveness appropriate for a biotype, though this likely arose from their awareness of the ambiguity inherent to the term. Apparently, in the definitions of Gallun and co-workers a single individual can justifiably be named as a biotype (“...a mating between a completely heterozygous Great Plains female and Great Plains male...would produce progeny having eight different biotypes.” (Gallun 1977, p. 227). Neilson et al. and Saxena and Barrion are more explicit that it is populations that are being differentiated, while Granett et al. seem to want to cover all bases and in so doing forfeit the game. “Shared phenotypic traits” without more detailed knowledge of their basis are notoriously vacant as criteria for classification (and naming a biotype is an act of classification), the definition of host race consigns it to a subcategory of an already dubious construct.

#### Clarification of associated terms

As noted above there are a number of terms that have been associated with the term biotype and their clarification may help to focus the discussion. For example, the term ‘strain’ is often synonymized with the term biotype. Whether they differ or not depends on the definition of biotype being used. Strain originated in the microbiological literature, denoting a culture derived from a given isolate and maintaining some distinctive trait. The laboratory cultures of many pest species to which the term biotype has been applied, such as the ‘purified’ stocks of the Hessian fly, might appropriately be called ‘strains’. They are thus artificial and it is not always clear how relevant they are to the situation in the field. The term ‘biological race’ was used in the older literature on host associated differentiation in phytophagous insects (Walsh 1864; Thorpe 1930; Smith 1940). These authors were primarily discussing what has come to be called ‘host races’, and both terms are essentially synonymous. The term host race itself has been used interchangeably with biotype. Granett et al. (2001) define host race as a special case of a biotype, where the “shared phenotypic trait” is an adaptation to a host that other members of the species are less well or not adapted to. This definition, however, is inconsistent with the now longstanding definition of a host race disseminated and advocated by Bush (1969), which is that a host race “is a population of a species that is *partially reproductively isolated* from other conspecific populations as a direct consequence of adaptation to a specific host” (Diehl and Bush 1984, p. 472). One could replace ‘host here with another factor involved in some degree of reproductive isolation. Fairly rigorous (though somewhat different) criteria have been outlined by Jaenike (1981) and Bush (1993) in order to demonstrate that a host race exists. Thus, a host race posits a mechanism of cohesion (partial reproductive isolation driven by natural selection through the host plant, though other factors besides selection could drive host race formation) that is entirely lacking in any conception of a biotype. It can be argued that biotype is a transitional concept used until more detailed data are available. Thus, once the criteria for host race formation are satisfied one can then drop the biotype name and rename the population as a host race, i.e., the ‘haw race’. But this practice merely clothes ignorance with baubles and bangles.

In the case of parthenogenetic insects such as aphids, a couple of choices are available for reference: individual, clone, and genotype. There is little to be gained by devising a new name (i.e., ‘biotype Z’).

In spite of this somewhat lengthy discussion of terminological nuances, it is a tenet of this review that at issue is not simply an argument about terminology but a conceptual conundrum. Some basic concepts in population genetics will now be reviewed that often seem to have been overlooked by many applied entomologists working with insects subject to phenotypic variation in host use.

### Basic population genetics of variation within and among populations

#### Variation within populations

Genetic variation among individuals within a population is the premier requirement for natural selection to operate and appears to be ubiquitous. It will be increased, or maintained, by input of new mutations, migration of genes from other populations, or by various forms of selection (balancing selection) that tend to prevent fixation of alleles. It will be decreased by reductions in population size and forms of selection (directional selection) that tend to promote fixation of alleles. Phenotypic variation is caused by genetic variation, but also by environmental influences; and a genetically homogenous population may present a range of phenotypes induced by differences in climate, resources, rearing environment, maternal condition, etc. Since selection is assumed to be strong where host plant resistance or other management tactics are involved, and is usually invoked in what is often called the ‘development of biotypes’, selection theory is a starting point to better understand how phenotypes might change over time.

Given an allele, *k*, that confers an increase in fitness (the phenotype ‘virulence’) on a given resistant host plant (or other tactic) at a frequency *q* within a population, an increase to fixation is assured as competing alleles decrease in frequency at a rate proportional to 1 — *hs* each generation, where *s* is the selection coefficient on a given genotype and *h* is the dominance coefficient. The source of the allele may be mutation or migration from another population (see below), but it may have been present in the population at very low frequency with no selective advantage prior to introduction of a management tactic. An elementary equation in population genetics states that for diploids the change in allele frequency is Δ*p* = *P*_t+1_ — *P*_t_ = *p(w_k_ -W) / W*, where *W_k_* is the fitness of a genotype bearing the allele and W is the mean fitness of the population (Crow and Kimura 1970). Obviously if *W_k_* > W, the allele increases in frequency until *p* = 1 (and *w* = W). This is the inexorable action of selection that justifies the concern over the spread of resistance breaking genotypes in many cropping systems. However, this theory applies to a single locus in a single randomly mating population. The deployment of resistant host plants in large monocultures applies the selection necessary to satisfy the equation, but does fixation (or progress toward fixation) of an allele within a population indicate the origin of a new entity or merit a taxonomic designation (biotype Z)? Well, it might — if more was known. For example, if fixation of allele *k* promoted assortative mating among individuals associated with the previously resistant host, either by linkage with mating preference genes or by ecological isolation, the birth of a new entity (race) might be underway. But the increase in frequency of a virulence allele (and its virulent phenotype) is just that — until more is known. Directional selection may be moving the population mean to a new position, but has something ‘new’ arisen? Reference to an entity called a biotype in this situation gives cohesion to that which has no cohesion and distinctiveness to that which is not distinct. Of course, a clone is a real entity, and if it happens to carry an allele for virulence and proliferates across the monoculture landscape the moniker biotype will seem justified — but a clone is a clone, an adequate and sufficient term to describe what it is.

Single-locus theory is the simplest way to see how alleles influencing traits such as fecundity or survival are affected by selection, but it is not likely that many of the life history traits that signify the success of pests and bane of crops are mono- or even oligogenic, in spite of the gene-for-gene paradigm invoked for systems such as the Hessian fly and wheat. Herein lies much of the potential for failure in predictive power embedded in the biotype concept because polygenic traits are subject to complex patterns of inheritance and expression. For example, the genes that condition host plant performance may be expressed differentially in different genetic backgrounds and in different environments (hosts). What this means, in an interbreeding population, is that a biotype may be here today, gone tomorrow — and back again the next day. The phenotypes, biotype Z and biotype X, will be a product of the segregation of a number of different gene combinations. Simple assays of performance on a small sample of hosts will suggest a simple distribution of variation (even bimodal) even though the underlying genetic variation may be continuous.

#### Mutation or migration?

When failures of management tactics such as host plant resistance occur the first hypothesis might be that a new mutation has occurred. However, the data on fitness effects of new spontaneous mutations in eukaryotes strongly suggest that a large majority of mutations are deleterious making it less plausible that increased fitness in a stressful environment (resistant hosts) will often come from new mutations (Lynch et al. 1999; Downie 2003). Of course, loss of function mutations in regulatory genes have been known to increase expression of genes for detoxifying enzymes against insecticides and may occur in the context of plant defensive compounds as well (Feyereisen 1999).

The other route to some low frequency of virulent alleles is of course migration. If one entertains this hypothesis for the origin of a virulent genotype it might be assumed that the migration event was recent. But this needn't be so, for low frequency alleles may reside within a large population (such as many pest populations) for a long time before either being lost or increasing in frequency if they are neutral, or being only slightly deleterious. This time will vary depending on the fitness effects, but also the dominance relationships of alleles at a locus as recessive alleles will persist longer than dominant alleles. In the environment of a given host in which allele *k* in a diploid population is neutral or only slightly deleterious, it may be maintained for 4N_e_ generations (N_e_ is the effective size of the population) before ultimately becoming fixed (though most will be lost much faster than this) (Kimura and Ohta 1969). In a sense it is ‘waiting’ for the environment in which it confers higher fitness on its bearer. Thus the increase in frequency of a virulent phenotype need not be initiated from new mutation *or* recent migration.

Entomologists that speak of the ‘development of biotypes’ often seem to be referring to the evolution of population differentiation however, a topic that is pursued below.

#### Variation among populations

Differences among spatially and/or ecologically distinct populations of the same species can be caused by genetic and environmental factors. Environmental causes of population differentiation must be ruled out before any inference of evolutionary change can be made. Genetic change leading to population differentiation could be caused by independent mutation accumulation between populations after an extrinsic restriction of gene flow, stochastic effects of small population size, or disruptive selection. The entomological literature using the biotype designation to denote differentiation of host-associated insects is concerned with disruptive selection as a cause of population differentiation, since it is the resistant host that is deemed to select for new biotypes. In other cases, such as virus transmission ability, the differentiation may have been stochastic under geographic isolation (or selection on the virus rather than the insect). An important complicating factor is that most pest species are introduced and have a history of host associated selection in their native range preceding introduction into managed habitats. Given the strong and stark selection insects are exposed to in managed systems one might expect host associated differentiation to occur more frequently in these than in unmanaged systems. However, the homogenous nature of cropping systems may make restriction in gene flow less likely than in the heterogeneous natural habitat and directional change in allele (or clonal) frequency the more likely outcome.

Population differentiation requires not just differentiation in the phenotype of host performance (and the loci that confer greater fitness on a host) which could sort onto hosts in the short term, but evidence for persistent restriction of gene flow across host associated populations. If such evidence is found, the criterion for race formation has been met and there is no need for the term biotype. Many molecular studies appear to have provided evidence for restricted or even absent gene flow, finding fixed differences or strongly supported clades, suggesting that races or cryptic species are involved. In some cases biotypes have been designated based on such findings apart from any response to management tactics (Perring 2001). Unfortunately many of these suffer from inadequate sampling or possible artifacts of laboratory reared ‘populations’. In the absence of better evidence there is little to be gained by applying the term biotype.

This requirement is impossible for clonal organisms (which constitute a large proportion of the insect species from which biotypes have been described). From a genetic standpoint one has to consider a clone as an individual; and the genetic differentiation observed between two different clones is merely individual level variation, not population differentiation. A clone could be considered a population with genetic variance of zero or near zero, but this ignores the fact that patterns of inheritance and the dynamics of change are very different from an interbreeding population. Variation amongst the component individuals of a clone is analogous to allelic variation within diploid (or polyploid) individuals. This variation is due to mutation only, but with recent estimates of genome wide mutation rates for quantitative characters in eukaryotes ranging from *U* = 0.02 to about 1.0 per generation, it is something that can be observed (Drake et al. 1998).

These points may seem moot from a purely management perspective but ignoring them sweeps the true genetic constitution, evolutionary history, and most importantly, the predicted evolutionary trajectory of the population under the rug.

### Examples of application of the biotype concept

#### The Hessian fly, *Mayetiola destructor* Say (Diptera: Cecidomyiidae)

It is appropriate to begin a discussion of examples of the usage of the biotype concept with *M. destructor* since one of the first applications of the term appears to have been by Painter (1941) working in the Hessian fly/wheat system. In this paper, and elsewhere, Painter (1951) used the terms biological races, strains, and biotype interchangeably. Previously Painter and others had discussed geographic populations that differed in virulence to wheat as biological races. Their thinking was thus clearly in the context of population differentiation, and they favored the hypothesis that selection by host plant traits promoted this differentiation. The change in terminology occurred without discussion in the literature. The meaning of biotype came to focus (expanded considerably since the 1990s) on the performance of a genotype or group of genotypes of unknown relation on particular hosts (host differentials) and has reached a refined level in *M. destructor* where currently there are some 16 named biotypes (though a number of these are laboratory creations). The classical genetic work of Hatchett and Gallun (1970) and Gallun (1977, 1978) and a study using allozymes by Black et al. (1990) clearly showed, however, that the phenotypes designated as ‘biotypes’ are equivalent to genotypes at loci affecting virulence that are segregating *within* populations and that the evolution of ‘biotypes’ in this system can be understood as a change in allele frequencies in randomly mating populations. This is in contradistinction to differentiation among genetically partially isolated host associated populations, though the distribution of allele frequencies differs among geographic populations (Ratcliffe et al. 1994, 1996). Further, Ratcliffe et al. (1994) and Ratcliffe et al. (1996) showed that allelic variants conferring virulence exist within populations prior to any selective pressure (as opposed to variants arising from new mutations or migration). What are being called biotypes are thus not discrete entities, and the more appropriate term ‘genotype’ should be employed (referred to by their allelic states if need be and if known). It is encouraging that in this system, where the biotype concept has the longest history, the trend has been toward abandoning it (Harris et al. 2003) due to the cumbersome and eventual accumulation of more named biotypes than letters of the alphabet (Patterson et al. 1992), but not because of the conceptual problems with the term itself.

It is somewhat surprising that fitness costs associated with virulence alleles, and *M. destructor* use of alternative hosts that might act as reservoirs for virulence alleles have been little studied, as noted by Harris et al. (2003).

#### The greenbug, *Schizaphis graminum* (Rondani) (Hemiptera: Aphididae)

As of 2006 (Burd and Porter 2006), nine biotypes of *S. graminum* had been specified. Burd and Porter extended this to 22. There is evidence that ‘biotypes’ can be produced from a round of sexual reproduction (Puterka and Peters 1989, 1995) and that some host associated genetic variation likely predates modern agriculture (Porter et al. 1997). Some evidence for host associated differentiation has been found (Shufran et al. 2000), but it is more inclusive than single biotypes and it is difficult to know how much of this differentiation exists in the field at the population level as samples were taken from long established laboratory clones. When populations in the field were sampled no correlation between biotype designation and mtDNA haplotype was found (Anstead et al. 2002). The evidence suggests that the various ‘biotypes’ are nothing more than recombinant genotypes segregating from periodic sexual reproduction, followed by asexual proliferation. As such they can be broken up and recreated with each round of sexual reproduction and bestowing a name or sub-taxonomic status upon them grants them a distinction which is unfounded. In this system researchers are essentially equating unknown genotypes at loci affecting host plant performance with biotypic designation. This has been recognized by those working on *S. graminum* but they continue to describe biotypes (Nuessly et al. 2008). That there is a geographic pattern to the distribution of the various genotypes is unsurprising. Such a pattern is to be expected in any variable environment with particular multi-locus genotypes going to fixation or near fixation in some regions and occurring at low frequencies elsewhere.

#### The rice brown planthopper, *Nilaparvata lugens* (Stal) (Hemiptera: Delphacidae)

The rice brown planthopper, *N. lugens* is a serious pest of rice and biotypes have been described since the mid-1970's (Chelliah and Bharathi 1993). As in other systems, detection of so-called biotypes and screening for resistance is done by assaying insects on different rice cultivars, with the assay insects often laboratory pure-bred and reared for long periods after originally being defined as biotype 1, 2, 3, 4, 5, etc. As in other systems, resistance thus seems to be designed to counter attack by artificial populations, a natural and insidious consequence of using the biotype concept. In the field responses of so-called biotypes were long ago shown to be variable (Claridge and Den Hollander 1980). Because resistance to *N. lugens* in rice has been controlled by major genes, it was assumed that the gene-for-gene paradigm fit this system, but this was shown to be incorrect by Den Hollander and Pathak (1981). Polygenic control of virulence means that many different genotypes can be created by segregation from a parental ‘biotype’. Though Claridge and Den Hollander as far back as 1983 cogently urged that the term biotype not be applied to this and other pest species, its use continues unabated (Naeemullah et al. 2009; Wu et al. 2009).

#### Grape phylloxera, *Daktulosphaira vitifoliae* Fitch (Hemiptera: Phylloxeridae)

Grape phylloxera, *D. vitifoliae,* is a pest of cultivated grapevines, and parthenogenetic reproduction predominates in these habitats. It is native to North America where it feeds on about six of the 18 or so species of North American *Vitis.* The collection and development of grapevines resistant to *D. vitifoliae* represents the first successful use of HPR against an insect pest, and HPR continues to be the primary management strategy. Early researchers suggested that different accessions of *D. vitifoliae* exhibited different levels of virulence to different grapevine cultivars; Börner (1914a, b), working in Europe in the early 1900's suggested that different species may be involved. Morphological distinctions between Börner's suggested division failed to be supported and the insect continues to be treated as a single species. Following Börner, the biotype concept was first applied to grape phylloxera in 1937 by Printz, and in 1970 by Stevenson, after which use of the term apparently became obligatory in papers regarding this insect. From 1970 to 1988, evidence was found for differential responses to resistant cultivars among grape phylloxera samples from New Zealand, Germany, South Africa, Ohio, and California. In only a single case (Williams and Shambaugh 1988) could any of these studies be said to have an explicitly genetic approach and that paper was flawed by a naive application. Most studies relied on assaying insect performance on excised root pieces, potted vines, or field vines from either pooled samples of grape phylloxera or a very small number of defined laboratory reared clones. It is not too surprising that different insect genotypes might perform differently on different plant genotypes. When the performance of individual genotypes is extrapolated to a population and the inference of population differentiation is made the limits of the data have been exceeded. A clonal organism like grape phylloxera presents the additional problem of defining what an individual is and what a population is. From the viewpoint of a grower with thousands of tiny insects damaging a crop there is no question: a large population plagues him. From the point of view of a population geneticist this large population is nothing but the elaboration of a single genotype; that this genotype expresses a phenotype different from another is not a manifestation of population differentiation. The change in frequency of genotypes within a population (increase of one clone over another) is not population differentiation but directional change. Nothing new has been formed, only the frequencies have changed. To the extent that the vague term biotype refers to a differentiated population its application in this situation is inappropriate and not useful.

When molecular markers began to be applied to the question of whether host-associated genetically-differentiated populations of grape phylloxera existed, it was found that the distribution of genetic variation did not correspond to the expectation (Fong et al. 1995; Downie et al. 2001, 2002). More recent studies suggest that host-associated differentiation may exist (Corrie et al. 2002), but these studies suffer from the problem noted above — clones, not populations are being differentiated by the markers and what is being documented is that different genotypes express different phenotypes, an unremarkable result.

#### The whitefly, *Bemisia tabaci* (Gennadius) (Hemiptera: Aleyrodidae)

Strictly speaking the *B. tabaci* species complex falls outside the scope of this review, as biotypes have been described and named based on a range of criteria other than host plant use such as insecticide resistance, esterase genotypes, virus transmission, and RAPD profiles (Perring 2001). The sheer chaos and disarray induced by use of the biotype concept makes its inclusion here imperative however. Early studies focused on host plant associated variation in these whiteflies (Bird 1957; Mound 1963; Costa & Russell 1975). Following the description of the B biotype (Costa & Brown 1991) searching for and describing *B. tabaci* biotypes became something of a cottage industry, with somewhere between 33 and 38 having been described to date (Xu et al. 2010; Dinsdale et al. 2010). The resultant grab bag of genetic variants forms an inconsistent, often localized, and disconnected set of hypotheses. It is not at all clear that this work has made much impact on management decisions or outcomes, with the possible exception of the so-called B biotype, controversially elevated to species status in 1994 (Bellows et al. 1994). It is interesting that a number of studies beginning in 1993 have shown reproductive incompatibilities between different collections of whitefly suggesting that races or species are more appropriate referents to these entities (Perring et al. 1993; Costa et al. 1993; Byrne et al. 1995; De Barro & Hart 2000; Maruthi et al. 2001, 2004; De Barro et al. 2005; Liu et al. 2007; Xu et al. 2010). Yet biotypes continued to be described and discussed. Recently the evidence for species status within the *B. tabaci* complex has been bolstered by Dinsdale et al. (2010) using a DNA barcoding method. Many of the described biotypes are subsumed within putative species of the complex in this study. Clearly the case of the *B. tabaci* complex is extremely complicated and difficult. It is rather less clear that the search for biotypes has done much to clarify the situation. Dinsdale et al. (2010, p. 206) make the point cogently: “This underscores the problem with assigning names to groups without considering their genetic bounds and suggests that most studies have taken place without a thorough consideration of the underlying genetic structure. As a consequence, biotype designations for *B. tabaci* have, in many cases, been confusing, lacking rigor and suggestive of the existence of significant biological variation where none might exist. Cessation of the use of biotype designations would remove this ambiguity and may aid in more accurate classifying and monitoring of distinct genetic groups.”

#### The fruits of the biotype concept: Has it led to a better understanding, or course of action against plant pests? Directions for the future

In all the cases described above, as well as examples from other systems and contexts other than HPR, it is clear that the naming, organization of thought, and design of studies around the concept of biotypes has produced more smoke than light. A range of disparate entities, non-entities, and phenomena are covered under its umbrella. The terms clone, genotype, strain, population, biological race, host race, and biotype have all been used with disconcerting equivalence. Use of such an umbrella term oversimplifies or disguises the true nature of the existing variation and so cannot effectively inform management decisions. For example, the practice of breeding resistant plants to specific strains or genotypes (often long term laboratory reared and homogenized), assumed to represent supposed biotypes, can only lead to failure since the assay conditions are not representative of a real population of insects in the field. It makes sense to study genetics on laboratory populations, but developing resistance against them seems less perspicacious. Durable resistance is an elusive goal, but simplistic perceptions of pest populations can only exacerbate the problem.

Whether a biotype corresponds to a cryptic species, a genetically differentiated population, or the now more common genotype from a directional change in allele frequencies within a population, an evolutionary phenomenon is invoked. Understanding this phenomenon dictates an explicit and informed evolutionary genetic approach be applied to the problem of adaptation of pest species to crop plants. The practice of naming or categorizing biotypes is vacuous and delusional and it would be heartening to think the last of it has been seen. It would appear that the practice has been on the decrease over the past decade or so, but an ISI Web of Science search found 38 insect studies in 2009 alone that applied the term, with and without names. A large proportion of these refer to the *B. tabaci* complex. While use of the term in a general manner prior to any useful research being carried out is not offensive, ideally even this application will be allowed to die a quiet death. This review thus echoes and strengthens the call given by Claridge and Den Hollander in 1983 to dispense with the term. It is a call for caution, restraint, consistency, and clarity. Variation in virulence to HPR (or other biological characters) should be treated in the context of the segregation of alleles and dynamics of gene frequencies (genotypic variation), and resistance should be developed and deployed against genetically variable populations not imagined homogenous ‘biotypes’. Only when partial or complete reproductive isolation can be demonstrated should populations of insects be treated as entities that are real and distinct (as races or species). The term biotype falls away.

## Abbreviation

HPR: host plant resistance
